# Intermittent Urine Oxygen Tension Monitoring for Predicting Acute Kidney Injury After Cardiovascular Surgery: A Preliminary Prospective Observational Study

**DOI:** 10.7759/cureus.16135

**Published:** 2021-07-03

**Authors:** Takao Kato, Yohei Kawasaki, Kaoru Koyama

**Affiliations:** 1 Department of Anesthesiology, Saitama Medical Center, Saitama Medical University, Kawagoe, JPN

**Keywords:** urine oxygen tension, cardiovascular surgery, cardiopulmonary bypass, acute kidney injury, kidney disease improving global outcomes classification

## Abstract

Introduction

Novel biomarkers of acute kidney injury (AKI) are being developed and commercialized. However, none are universally available. The aim of this preliminary prospective observational study was to explore the effectiveness of intermittent urine oxygen tension (PuO_2_) monitoring without special equipment (using a blood gas analyzer) for predicting AKI after elective cardiovascular surgery requiring cardiopulmonary bypass (CPB).

Methods

Fifty patients who underwent elective cardiovascular surgery requiring CPB were enrolled in the study with written informed consent. Urine samples were intermittently collected from a urethral catheter at four points: T1, immediately after induction of general anesthesia in the operating room; T2, immediately after intensive care unit (ICU) admission; T3, six hours after ICU admission; and T4, 12 hours after ICU admission. PuO_2_ was measured with a blood gas analyzer. The Kidney Disease Improving Global Outcomes classification was used for the diagnosis of AKI, then patients were followed up until postoperative day 7. By generating the receiver operating characteristic curves, the cut-off value of PuO_2_ and area under the curve (AUC) for predicting the onset of AKI was calculated. The odds ratio (OR) and 95% confidence interval (CI) of each time point were calculated using logistic regression analysis or exact logistic regression method. P < 0.05 was considered significant.

Results

Twelve patients were diagnosed with AKI (24% morbidity). The cut-off values of PuO_2_ for predicting onset of AKI at the four time points were T1, PuO_2_ ≥ 132.4 mmHg (OR 3.1, 95% CI 0.78-12.0, p = 0.11, AUC 0.57); T2, PuO_2_ ≥ 153.3 mmHg (OR 5.8, 95% CI 1.08-31.4, p = 0.04, AUC 0.51); T3, PuO_2_ ≥ 130.1 mmHg (OR 0.19, 95% CI 0.05-0.75, p = 0.018, AUC 0.68); T4, PuO_2_ ≥ 88.6 mmHg (OR 0.07, 95% CI 0-0.486, p = 0.011, AUC 0.64).

Conclusion

Intermittent PuO_2_ values at six and 12 hours after ICU admission may be predictors of AKI, although the AUCs to predict AKI were low (0.68 and 0.64). AKI prediction by PuO_2_ was not possible immediately after induction of general anesthesia (not statistically significant) and immediately after ICU admission (AUC was very low). Further studies are required to confirm the validity of intermittent PuO_2_ monitoring.

## Introduction

Perioperative organ injury is a leading contributing factor to morbidity and mortality in surgical patients [1]. Among such injuries, acute kidney injury (AKI) has an extremely harmful effect on surgical outcomes [2]. Serum creatinine and urine output are currently used as the gold standard for diagnosing AKI in criteria, such as RIFLE (Risk, Injury, Failure, Loss, End-stage renal disease) [3], AKIN (Acute Kidney Injury Network), modification of RIFLE [4], and KDIGO (Kidney Disease Improving Global Outcomes) criteria [5, 6]. However, serum creatinine is a late, insensitive, and nonspecific indicator of AKI [7, 8]. Numerous clinical trials have examined the sensitivity and specificity of novel acute kidney biomarkers [9-14], none have been used as worldwide diagnostic criteria for AKI.

Hypoxia of the renal medulla has been found to be a feature in AKI due to cardiopulmonary bypass (CPB) and sepsis [15-17]. Urine oxygen tension (PuO_2_) of the bladder was highly correlated with oxygen tension of the renal medulla in animal studies [18-20]. Continuous monitoring of PuO_2_ using a fiber optic probe at the tip of the urinary catheter during cardiac surgery requiring CPB may be effective for predicting AKI in humans [21, 22].

Sensitive AKI biomarkers are not available all over the world, and continuous PuO_2_ monitoring requires a special probe. On the other hand, intermittent measurement of PuO_2_ is possible at any center in the world by collecting urine through a urinary catheter and using a blood gas analyzer. Therefore, the purpose of this preliminary study was to investigate the possibility of predicting perioperative AKI by intermittent PuO_2_ measurement.

## Materials and methods

Study design

A single-center, prospective observational study was conducted in accordance with the good clinical practice and the guidelines set out in the Declaration of Helsinki. The study was approved by the local Ethics Committee (authorization number: 1753-Ⅲ) and written informed consent was obtained from all patients. The Strengthening the Reporting of Observational studies in Epidemiology (STROBE) Checklist was used.

Setting, participants and study size

Patients scheduled for elective cardiac surgery requiring CPB at a university hospital between January 2018 and December 2019 were enrolled in the study. Patients meeting any of the following criteria were excluded: estimated glomerular filtration rate (eGFR) <60 ml/min/1.73 m^2^ (including hemodialysis), history of urinary tract modification surgery, concurrent noncardiac surgery, and unable to provide informed consent. The eGFR was calculated by the equation devised by the Japanese Society of Nephrology [eGFR (mL/min/1.73 m^2^) = 194*Serum creatinine^-1.094^*Age^-0.287^*0.739 (if female)] [23]. Patients were followed up until postoperative day 7.

Due to the exploratory nature of the study and the difficulty of establishing a sample size using statistical methodology, the target number of patients was tentatively set at 50. As a result, the study period was two years.

Anesthesia and perfusion

Anesthesia was induced with intravenous administration of midazolam (0.02-0.1 mg/kg), fentanyl (1-2 μg/kg), and rocuronium (0.6-1 mg/kg), and maintained with sevoflurane (0-2%), propofol (1-6 mg/kg/h), remifentanil (0.02-0.2 μg/kg/min), fentanyl and rocuronium. An invasive arterial pressure line (FloTrac^TM^ Sensor, Edwards Lifesciences, Irvine, CA) was inserted into the radial artery, and a central venous catheter (Edwards Oximetry^TM^ CV catheter, Edwards Lifesciences) was inserted in the internal jugular vein. The arterial pressure, cardiac index (APCI), stroke volume variation (SVV), central venous pressure (CVP), and central venous oxygen saturation (ScvO_2_) were monitored as hemodynamic parameters. Red blood cell transfusions were performed with a target Hb >8.0 g/dL.

CPB was established by cannulation of the ascending aorta and of the superior and inferior vena cava. Heparin was administered to achieve an activated clotting time above 480 s. The CPB circuit included an arterial line filter (Filtia^TM^ FT-50, JMS, Tokyo, Japan), a roller pump (S5^TM^, Livanova, London, UK), and a hard-shell membrane oxygenator (Oxia^TM^ ACF, JMS), and was primed with a mixed solution (1000 ml of lactate Ringer solution, 300 ml of D-mannitol, 100 ml of 25% albumin and 6000 IU of heparin). Non-pulsatile pump flow of 2.2-2.6 L/min/m^2^, hematocrit of >23%, and venous oxygen saturation of >70% were maintained as target. Body temperature was maintained between 28 and 32℃ (25℃ in circulatory arrest) using a standard heater-cooler system (3T^TM^, Livanova). Cardiac arrest was induced and maintained by intermittent anterograde or retrograde cardioplegia with mixed infusion solution (500 ml of lactate Ringer solution, 40 ml of potassium chloride, 40 ml of sodium bicarbonate and 8.3 mg of diltiazem) and patient blood at a ratio of 4:1. After weaning from CPB and removal of the cannula, heparin was reversed with protamine. In addition to patient characteristics (activated clotting time, platelet count, antiplatelet medication, or liver failure) and clinical signs (surgical bleeding), rotational thromboelastometry (ROTEM^TM^ Sigma; IL Werfen, Munich, Germany) was used to evaluate the need for fresh frozen plasma and platelet transfusions, and the amount of protamine.

Intermittent measurement of PuO_2_

After induction of general anesthesia, a urinary catheter with a temperature sensor (Safeed^TM^ Silicone Balloon Catheter, Terumo, Tokyo, Japan) was inserted. The catheter was connected to a system of standard urine collection. Urine samples were anaerobically collected from a urine sampling port using a 1-ml syringe with a needle. PuO_2_ was measured with a blood gas analyzer (Stat Profile pHOx Ultra^TM^ Blood Gas Analyzer, Nova Biomedical, Waltham, MA, USA) at the following four points: T1, immediately after induction of general anesthesia in the operating room; T2, immediately after intensive care unit (ICU) admission; T3, six hours after ICU admission; T4, 12 hours after ICU admission. In the present ICU, approximately 80% of the patients who were potentially eligible for the study left the ICU within 12 hours after admission, and PuO_2_ measurement could not be continued in the general ward because blood gas analysis could not be performed immediately after urine collection. We did not measure the data after 12 hours of ICU admission because the number of patients who could be measured was too small to allow comparison.

Definition of AKI

Serum creatinine (sCr) was measured preoperatively, at admission to the ICU, and six and 12 hours after admission to the ICU. Thereafter, sCr was measured every 12 hours until peak out and every 24 hours after peak out for seven days after surgery. The baseline for sCr was defined as the last value before surgery. Urine output continued to be measured by storing urine for seven days after ICU discharge and removal of the urethral catheter. AKI was defined by KDIGO criteria: increase in sCr by ≥0.3 mg/dL within 48 hours; or increase in sCr to ≥1.5 times baseline within seven days after surgery; or urine output <0.5 ml/kg/h for six hours [5, 6].

Outcomes, confounders and bias

The primary outcome was the ability to predict AKI at each of the four time points. Systemic oxygen tension (PaO_2_) and urine output were considered as potential factors affecting PuO_2_ [21]. Then PuO_2_, PaO_2_, and urine output were compared at each time point between no AKI and AKI groups as the secondary outcomes. Urine output of T1 was calculated using the urine output from the insertion of the urethral catheter to the start of cardiopulmonary bypass, and the urine output of T2 was calculated using the urine output from the start of cardiopulmonary bypass to ICU admission. The urine volume at T1 reflects the effect of general anesthesia, and the urine volume at T2 reflects the effect of CPB. This study was small and exploratory, and no adjustment for bias was made.

Statistical analysis

Continuous data are presented as mean ± standard deviation, and categorical data as number and percentage. A Student t-test was used to evaluate continuous variables, and a Fisher exact test was used for categorical variables. By generating the receiver operating characteristic (ROC) curves, the cut-off value for PuO_2_ in predicting the onset of AKI was calculated at the four time points. The area under the curve (AUC) and Youden index was used to assess the ability to distinguish between no AKI and AKI patients. The odds ratio (OR) and 95% confidence interval (CI) at each time point were calculated using logistic regression or exact logistic regression method. P < 0.05 was considered significant. All calculations were performed using JMP^TM^ Pro ver.14.0 for Windows (SAS Institute, Cary, NC, USA).

## Results

**Figure 1:**
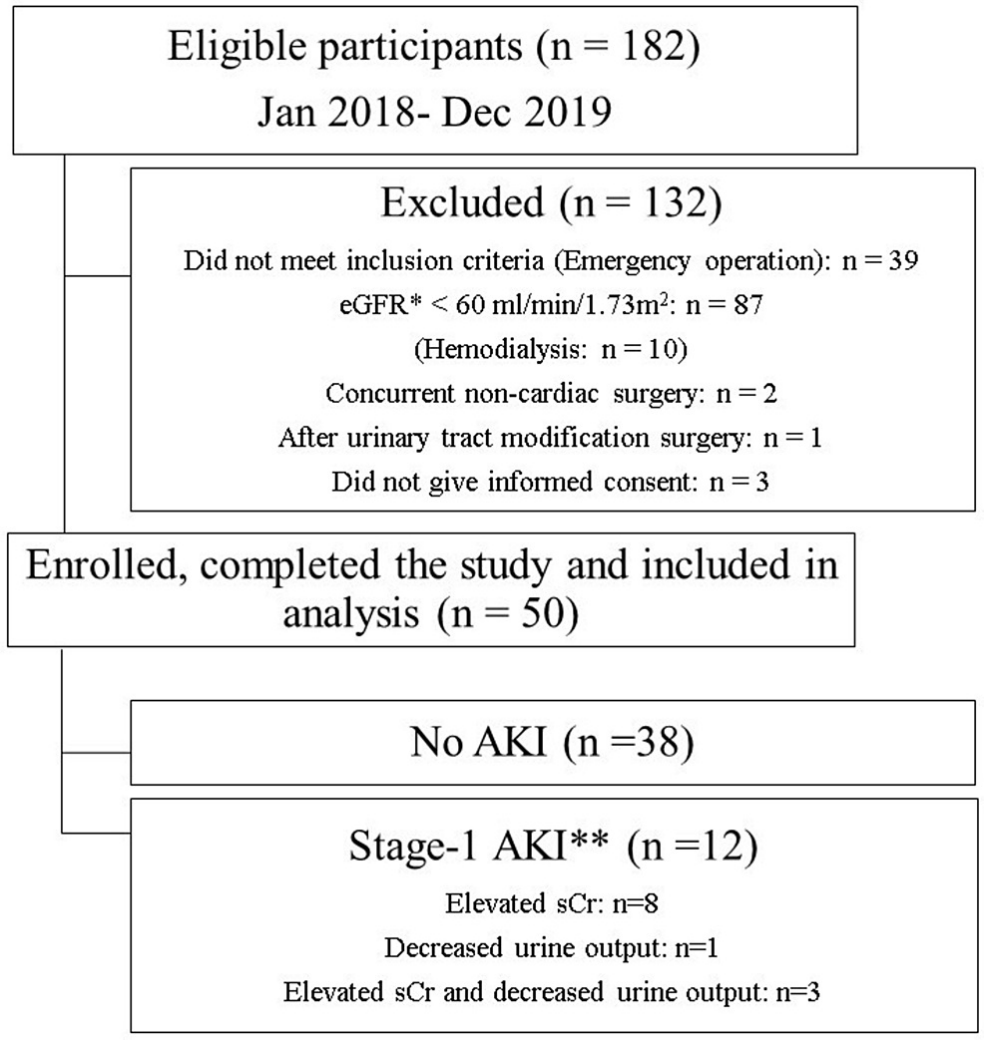
Flowchart eGFR: estimated glomerular filtration rate; AKI: acute kidney injury; sCr: serum creatinine * eGFR was calculated by the equation devised by the Japanese Society of Nephrology [23]. ** Definition of stage 1 AKI: sCr, increase of ≥0.3 mg/dL within 48 hours or 1.5 to 1.9 times baseline; or urine output, <0.5 ml/kg/h in 6 to 12 hours

Of the 182 patients who underwent cardiovascular surgery requiring CPB between January 2018 and December 2019, 132 were excluded. Fifty patients were enrolled and completed the study (Figure 1).

A total of 200 PuO_2_ values were collected (50 values at each of the four time points). Twelve patients (24%) were diagnosed with stage 1 AKI (eight based on creatinine criteria, one on the urine criteria, and three on both). No patients had stage 2 or 3 AKI, none required dialysis, and there were no perioperative deaths. The timing of AKI diagnosis was, on average, 23 ± 8.0 hours after admission to the ICU. The patient characteristics at baseline are shown in Table 1 and intraoperative and perfusion data are shown in Table 2. CPB time (202 ± 79 vs 157 ± 41) and Aorta clamp time (135 ± 67 vs 101 ± 44) were significantly longer in AKI group compared to no AKI group.

**Table 1:** Patient characteristics Continuous data are presented as mean ± standard deviation, and categorical data as number (percentage). LVEF: left ventricular ejection fraction; sCr: serum creatinine; eGFR: estimated glomerular filtration rate; CPB: cardiopulmonary bypass; CABG: coronary artery bypass grafting; ACE-I: angiotensin-converting enzyme inhibitor; ARB: angiotensin receptor blocker; ASA-PS: American Society of Anesthesiologists physical status; EuroSCORE: European System for Cardiac Operative Risk Evaluation. * eGFR was calculated by the equation devised by the Japanese Society of Nephrology [23].

Variable	All patients (n = 50)	No AKI (n = 38)	AKI (n = 12)	P value
Age (years)	68 ± 11	69 ± 12	67 ± 8.7	0.53
Gender (male, %)	28 (56%)	18 (47%)	10 (83%)	0.03
Weight (kg)	59 ± 13	56 ± 11	67 ± 15	0.02
Height (cm)	158 ± 10	154 ± 10	160 ± 9.2	0.36
Body Mass Index (kg/m^2^)	23 ± 3.6	23 ± 5.8	26 ± 6.7	0.01
Preoperative LVEF (%)	58 ± 15	59 ± 15	55 ± 16	0.39
Preoperative sCr (mg/dL)	0.73 ± 0.12	0.69 ± 0.12	0.79 ± 0.11	0.03
Preoperative eGFR^*^ (ml/min/1.73 m^2^)	74 ± 13	73 ± 14	72 ± 10	0.61
Type of surgery				
CABG	27 (54%)	21 (55%)	6 (50%)	1.00
Valve surgery	19 (38%)	14 (37%)	5 (42%)	
Thoracic aortic surgery	4 (8.0%)	3 (7.9%)	1 (8.3%)	
Comorbidity				
Diabetes mellitus	20 (40%)	14 (37%)	6 (50%)	0.50
Preoperative insulin	3 (6.0%)	2 (5.3%)	1 (8.3%)	1.00
Hypertension	38 (76%)	27 (71%)	11 (92%)	0.25
Preoperative ACE-I or ARB	17 (34%)	13 (34%)	4 (33%)	1.00
Dyslipidemia	26 (52%)	18 (47%)	8 (67%)	0.32
Smoking history	26 (52%)	20 (53%)	6 (50%)	1.00
Chronic lung disease	5 (10%)	5 (13%)	0 (0%)	0.31
Cerebrovascular disease	16 (32%)	8 (21%)	8 (67%)	0.01
ASA-PS classification (3, %)	50 (100%)	38 (100%)	12 (100%)	1.00
Logistic EuroSCORE (%)	5.3 ± 4.1	4.9 ± 4.4	5.7 ± 3.0	0.79

**Table 2:** Intraoperative and perfusion data Data are shown as mean ± standard deviation, and categorical data as number (percentage). CPB: cardiopulmonary bypass; P_A_O_2_: systemic oxygen tension during CPB; P_A_CO_2_: systemic carbon dioxide tension during CPB; MAP: mean arterial pressure; CVP: central venous pressure; SvO_2_: mixed venous oxygen saturation.

Variable	All patients (n = 50)	No AKI (n = 38)	AKI (n = 12)	P value
Operation time (min)	325 ± 70	311 ± 57	367 ± 91	0.01
Operation - CPB time (min)	155 ± 42	154 ± 43	165 ± 42	0.45
Anesthesia time (min)	423 ± 82	406 ± 75	478 ± 85	0.007
Anesthesia - CPB time (min)	259 ± 45	249 ± 51	276 ± 41	0.11
Intraoperative blood transfusion	15 (30%)	10 (26%)	5 (41%)	0.47
Pump flow (L/min)	3.8 ± 0.5	3.8 ± 0.5	4.0 ± 0.6	0.21
Pump flow (L/min/m^2^)	2.4 ± 0.2	2.5 ± 0.5	2.5 ± 0.5	0.30
P_A_O_2_ (mmHg)	271 ± 26	273 ± 28	268 ± 19	0.82
P_A_CO_2_ (mmHg)	39 ± 4.5	39 ± 5.0	40 ± 2.2	0.60
Hemoglobin (g/dL)	9.5 ± 1.0	9.4 ± 1.0	9.6 ± 0.9	0.89
Hematocrit (%)	28 ± 3.0	29 ± 3.0	28 ± 3.0	0.81
CPB time (min)	168 ± 55	157 ± 41	202 ± 79	0.01
Aorta clamp time (min)	109 ± 52	101 ± 44	135 ± 67	0.04
Mean MAP (mmHg)	53 ± 9.6	52 ± 10	55 ± 6.9	0.39
Mean CVP (mmHg)	2.3 ± 1.5	2.4 ± 1.7	2.0 ± 0.9	0.74
Mean SvO_2_ (%)	86 ± 6.2	86 ± 7.1	87 ± 5.5	0.81
Bottom temperature (℃)	30 ± 2.7	30 ± 2.7	30 ± 2.6	0.62

**Figure 2:**
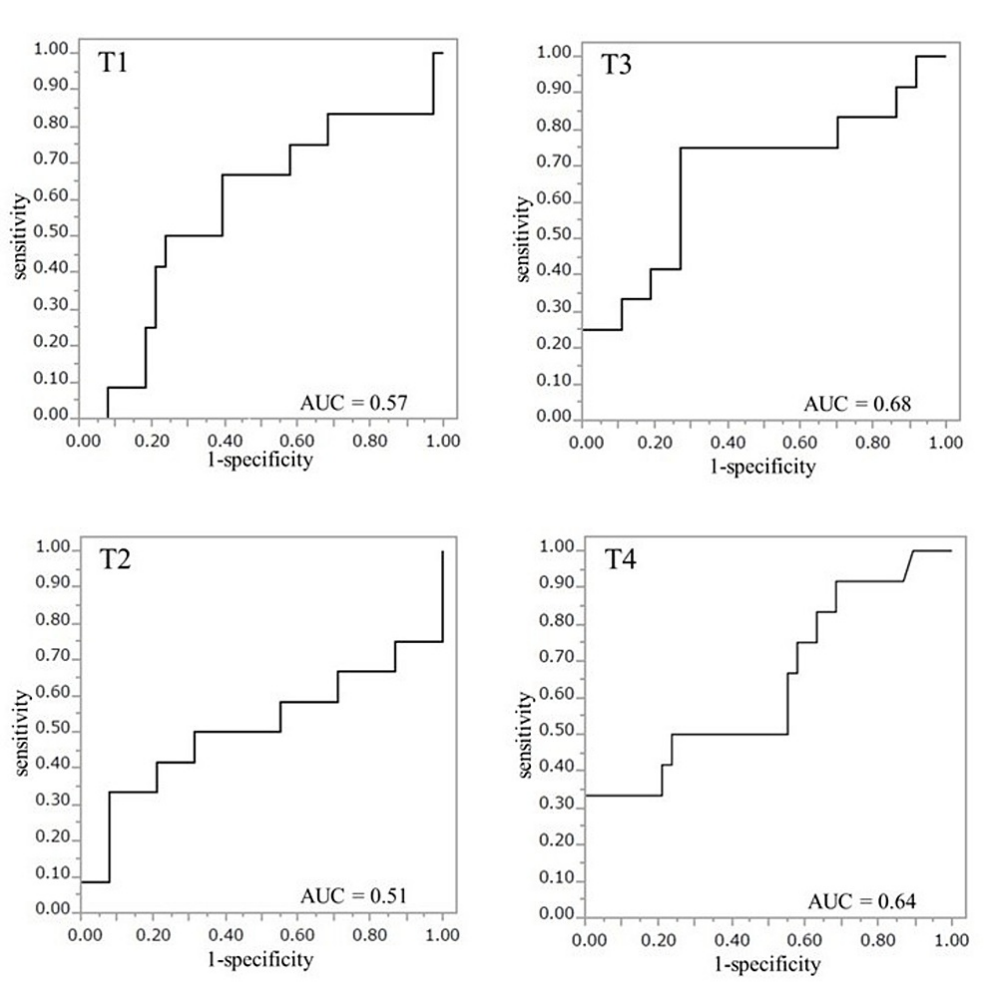
The receiver operating characteristic curve for T1, T2, T3, and T4. Time points: T1, immediately after induction of general anesthesia in the operating room; T2, immediately after intensive care unit (ICU) admission; T3, 6 hours after ICU admission; T4, 12 hours after ICU admission. AUC: area under the curve

The cut-off values of PuO_2_ for predicting onset of AKI at the four time points were T1, PuO_2_ ≥ 132.4 mmHg (OR 3.1, 95% CI 0.78-12.0, p = 0.11, AUC 0.57); T2, PuO_2_ ≥ 153.3 mmHg (OR 5.8, 95% CI 1.08-31.4, p = 0.04, AUC 0.51); T3, PuO_2_ ≥ 130.1 mmHg (OR 0.19, 95% CI 0.05-0.75, p = 0.018, AUC 0.68); T4, PuO_2_ ≥ 88.6 mmHg (OR 0.07, 95% CI 0-0.486, p = 0.011, AUC 0.64) (Figure 2).

Perioperative PuO_2_, PaO_2_ and urine output at the four time points are shown in Table 3.

**Table 3:** Urine oxygen tension, systemic oxygen tension and urine output at the four measurement time points Time points: T1, immediately after induction of general anesthesia in the operating room; T2, immediately after intensive care unit (ICU) admission; T3, 6 hours after ICU admission; T4, 12 hours after ICU admission. Data are shown as means ± SD. * Urine output of T1 was calculated using the urine output from the insertion of the urethral catheter to the start of cardiopulmonary bypass, and the urine output of T2 was calculated using the urine output from the start of cardiopulmonary bypass to ICU admission. PuO_2_: urine oxygen tension; PaO_2_: systemic oxygen tension

Variable	All patients (n = 50)	No AKI (n = 38)	AKI (n = 12)	P value
PuO_2_ (mmHg)				
T1	128 ± 29	126 ± 29	133 ± 31	0.45
T2	122 ± 24	122 ± 19	124 ± 37	0.80
T3	132 ± 26	136 ± 22	118 ± 33	0.03
T4	128 ± 29	132 ± 26	116 ± 33	0.10
PaO_2_ (mmHg)				
T1	214 ± 97	208 ± 99	234 ± 90	0.42
T2	154 ± 49	153 ± 47	160 ± 58	0.66
T3	125 ± 29	126 ± 31	123 ± 25	0.72
T4	109 ± 25	107 ± 19	116 ± 39	0.32
Urine output* (ml/min)				
T1	1.0 ± 0.8	1.1 ± 0.9	0.7 ± 0.5	0.14
T2	1.4 ± 1.2	1.5 ± 1.3	1.1 ± 0.8	0.30
T3	1.4 ± 0.6	1.5 ± 0.6	1.2 ± 0.5	0.11
T4	2.1 ± 0.8	2.3 ± 0.8	1.6 ± 0.4	0.01

The mean value of PuO_2_ was around 120-130 mmHg, and the mean value of urine volume was around 1-2 ml/min. In the comparison between no AKI and AKI groups, there were significant differences in PuO_2_ at T3 and urine volume at T4.

## Discussion

The primary outcome of this preliminary study indicated that low PuO_2_ at T3 and T4 may be a predictor of AKI, although the AUCs to predict AKI were low (T3, 0.68; T4, 0.64). AKI prediction by PuO_2_ was not possible at T1 (not statistically significant) and T2 (AUC was very low). From the secondary outcome, no significant difference was observed in PuO_2_ between no AKI and AKI patients except in T3, and the PuO_2_ values were much higher than in previous studies. The urine output was much lower than in previous studies, which could be related to the high PuO_2_ values.

Based on the KDIGO criteria, sCr was followed up to postoperative day 7. PuO_2_ was measured only up to 12 hours after admission to the ICU, because approximately 80% of the potentially eligible patients were discharged from the ICU on the first postoperative day. However, we thought that following up to 12 hours after admission to the ICU would be sufficient because PuO_2_ is lowest during CPB and then recovers slowly, as shown in previous studies [21, 22]. The mean time of diagnosis of AKI by KDIGO criteria was 23 ± 8.0 hours after ICU admission in our study and 20.6 ± 14.8 hours in the study by Zhu et al. [22], indicating that intermittent PuO_2_ may diagnose AKI slightly earlier than KDIGO criteria.

All previous studies have demonstrated that low PuO_2_ levels are at risk for AKI [18-22] and similar results were obtained in the present study. However, the ORs for T1 and T2 were reversed from those for T3 and T4, indicating a trend toward higher PuO_2_ being a risk for AKI at T1 and T2, although the AUC was low and not statistically significant for T1. Urine flow was extremely low immediately after induction of anesthesia (1.0 ± 0.8 ml/min) and immediately after ICU admission (1.4 ± 1.2 ml/min), and systemic PaO_2_ was higher during surgery (214 ± 97 and 154 ± 49 mmHg) (Table 3). Therefore, increased exposure of urine to air and the bladder wall may have increased PuO_2_. However, after ICU admission, low PuO_2_ levels may reflect a risk of AKI due to increased urine output (1.4 ± 0.6 and 2.1 ± 0.8 ml/min) and lower systemic PaO_2_ (125 ± 29 and 109 ± 25 mmHg).

In animal studies, the baseline for PuO_2_ is 35-50 mmHg [18-20], but in humans this value differs between reports: 89 ± 22 mmHg in Kainuma et al. [21], 65.5 ± 34.5 mmHg in Zhu et al. [22], and 50 ± 1.8 mmHg in Hong et al. [24]. Insertion of a urethral catheter can introduce air into the bladder, and PaO_2_ in the bladder wall is the same as systemic PaO_2_. Thus, elevated PuO_2_ at baseline may be due to diffusion of oxygen from air that has strayed into the bladder and the bladder wall, in accordance with Fick's law. Furthermore, the proximal end of the urine collection site in our study suggests that diffusion of oxygen from the air in the bag can cause a large increase in PuO_2_. The oxygen tension of the liquid is expected to approach 150 mmHg when the diffusion of oxygen in contact with air reaches equilibrium by Henry’s and Fick’s law [(atmospheric pressure - water vapor pressure at 37°C)×F_I_O_2_: (760-47)×0.21≒150]. The PaO_2_ of a small amount of bovine blood mixed with a saline solution equilibrated under air has been reported to be 165.8 ± 1.2 mmHg [25].

PuO_2_ is not constant unless the urine volume is >6 ml/min [24]. In previous studies, urine flow was relatively high: 3-8 ml/min in Kainuma et al. [21] and 4-6 ml/min in Zhu et al. [22]. In contrast, urine flow was 1-2 ml/min in our study. Therefore, PuO_2_ values may be higher due to greater exposure of urine to air and the bladder wall. Ngo et al. have suggested that the confounding factors for PuO_2_ are high systemic PaO_2_ and low urine volume [26].

Numerous clinical trials have examined the sensitivity and specificity of novel acute kidney biomarkers, such as neutrophil gelatinase-associated lipocalin (NGAL) [9], kidney injury molecule-1 (KIM-1) [10], cystatin C [11], tissue inhibitor of metalloproteninases-2 (TIMP-2), and insulin-like growth factor binding protein-7 (IGFBP-7) [12]. Among them, urinary TIMP-2 and IGFBP-7 have shown as the most valuable [13]. The NephroCheck^TM^ (Astute Medical, San Diego, CA, USA) that calculates the risk of renal injury using the product of urinary TIMP-2 and IGFBP-7 was authorized by the Food and Drug Administration in 2014 and achieved high sensitivity while maintaining acceptable specificity [14]. However, none have been used as worldwide diagnostic criteria for AKI. Zhu et al. have shown that continuous PuO_2_ measurement can predict postoperative AKI at intraoperative time points according to the severity and duration of the PuO_2_ decrease [22]. Intermittent PuO_2_ measurement cannot measure the duration of PuO_2_ decrease, and thus is considered inferior to the diagnostic capability of continuous PuO_2_. However, it is possible to capture the severity of PuO_2_ decrease by increasing the number of measurement points, especially during CPB, and this can be done worldwide using blood gas analyzers.

There are several limitations in the study, in part because it is a small preliminary observational study. First, no special equipment was used, and exposure to large amounts of air at the urine collection site leads to a high PuO_2_. Furthermore, it is most important to collect urine during CPB because PuO_2_ is the lowest, as reported by Zhu et al. [22]. Although the present study was not adjusted for confounders, CPB time and Aorta clamp time were significantly longer in AKI group compared to no AKI group, which is consistent with the study by Zhu et al. [22] and suggests that CPB is associated with the development of AKI. However, we could not collect urine during surgery and CPB because the site where we could collect urine cleanly from the urinary catheter was very close to the patient and would interfere with the surgical operation. Since this was an observational study, the timing of sCr measurements was also limited, and the timing of urine collection was set to coincide with the timing of postoperative sCr measurement, except after induction of anesthesia. A bladder catheter that allows for anaerobic and clean urine samples to be collected from inside the bladder at any time may increase the generalizability of the results. Second, due to the unexpectedly high PuO_2_ values, the number of cases required exceeded the number of patients enrolled (n = 50). Therefore, the cut-off for PuO_2_ at each time point might not be accurately assessed. Third, although chronic kidney disease was one of the risk factors of postoperative AKI [7], maintaining urine output was important in this preliminary study (PuO_2_ measurements cannot be made in anuric patients). However, forced diuresis may lead to progression of renal damage [27]. Therefore, we had to exclude the patients impaired renal function (eGFR < 60 ml/min/1.73 m^2^). Fourth, the validity of measurements on the urine samples is not certain because a blood gas analyzer for whole blood measurement was used, although this should be an acceptable method.

## Conclusions

This preliminary study examined the cutoff value of PuO_2_ in predicting AKI and indicates that PuO_2_ ≥130.1 mmHg (OR 0.19, AUC 0.68) at six hours after ICU admission and PuO_2_ ≥ 88.6 mmHg (OR 0.07, AUC 0.64) at 12 hours after ICU admission may be a predictor of AKI. However, the AUCs are low and still insufficient to predict AKI. In order to improve the accuracy of predicting AKI, it is necessary to develop a urine collection method that allows anaerobic urine collection at any time point and to increase the timing of urine collection especially during CPB.
